# Effects on impulsivity and delay discounting of intermittent theta burst stimulation add-on to dialectical behavioral therapy in borderline personality disorder: a randomized, sham-controlled pilot trial

**DOI:** 10.1186/s40479-025-00278-3

**Published:** 2025-01-14

**Authors:** Milenko Kujovic, Christian Bahr, Mathias Riesbeck, Daniel Benz, Martina Deiß, Zsofia Margittai, Sebastian Henges, Dirk Reinermann, Christian Plewnia, Eva Meisenzahl

**Affiliations:** 1 Department of Psychiatry and Psychotherapy, Medical Faculty, University Hospital Düsseldorf, Heinrich-Heine-University Düsseldorf, Düsseldorf, Germany; 2 Department of Psychiatry and Psychotherapy, University Hospital of Psychiatry and Psychotherapy, Tübingen, Germany

**Keywords:** DBT, rTMS, iTBS, Impulsivity, Decision-making, Delay discounting

## Abstract

**Background:**

Dialectical behavioral therapy (DBT) and repetitive transcranial magnetic stimulation (rTMS) are both effective in treating borderline personality disorder (BPD). Impulsivity and impaired decision-making are prominent features of BPD, and therapeutic interventions targeting these symptoms could lead to significant improvements.

**Objective/Hypothesis:**

We hypothesized that intermittent theta burst stimulation (iTBS), a modified rTMS protocol that targets the left dorsolateral prefrontal cortex, would enhance the therapeutic effects of DBT, leading to greater improvements in impulsivity and decision-making compared with sham stimulation.

**Methods:**

We performed a single-blind, randomized, sham-controlled pilot study to evaluate the efficacy of iTBS as an add-on to an 8-week DBT program for BPD in a routine inpatient setting. A total of 53 BPD patients were randomly assigned to receive either iTBS (*n* = 25) or sham stimulation (*n* = 28) during weeks 4 to 8 of DBT, and 36 patients met the inclusion criteria for the present analysis (*≥* 16 of 20 iTBS/sham sessions and assessment of delay discounting). The study endpoints were the Barratt Impulsiveness Scale-15 for impulsivity and the Monetary Choice Questionnaire for decision-making/delay discounting.

**Results:**

A mixed model repeated measures analysis with a 2 × 2 factorial between-subjects design showed a significant overall improvement over time in impulsivity but not in decision-making/delay discounting. No significant differences were found between iTBS and sham, although post hoc tests revealed significant changes in impulsivity in the iTBS group (mean_diff_ = -4.7, *p* = .001, Cohen’s *d* = 0.68) but not in the sham group (mean_diff_ = -2.1, *p* = .077, *d* = 0.31).

**Conclusions:**

iTBS may offer long-term benefits as an add-on treatment to DBT for impulsivity in BPD, suggesting the need for further investigation in larger-scale studies.

**Trial registration:**

Registered at drks.de (no. DRKS00020413) on January 13, 2020.

**Supplementary Information:**

The online version contains supplementary material available at 10.1186/s40479-025-00278-3.

## Background

Borderline Personality Disorder (BPD) is a complex mental disorder that is commonly associated with emotional instability, altered interpersonal relationships, impulsivity, and self-harming and suicidal behavior [1]. The prevalence of BPD ranges from 0.7 to 2.7% in the general population and is approximately 22% in patients in psychiatric hospitals [1].

Impulsivity is of particular significance in BPD because it is associated with potentially self-harming behavior, such as substance abuse, reckless driving, and monetary expenditure, as well as physical self-harm and suicidal behavior, and accordingly, it is commonly defined as a symptom of the disorder [2, 3]. Furthermore, as Sebastian et al. [4] stated, from a broader perspective impulsivity is a complex clinical concept consisting of several aspects that are differently associated with BPD. For example, impulsivity can also be viewed as a facet of emotional dysregulation [4]. Consequently, no clear definition of impulsivity exists; however, key features include a proneness towards certain behaviors, a lack of foresight, and failure to anticipate the (long-term) outcomes of behavior [3].

Decision-making can be defined as choosing a specific action from various alternative options with the anticipation of yielding the most advantageous outcome for the decision-maker [5]. Accordingly, impulsivity and decision-making are closely related and are often assessed with similar methods [6]. Thus, symptoms of impulsivity in BPD (e.g., drug use, impulsive spending, sexuality, binge eating) may be reflected in decision-making, with an imbalance of short-term, tension-relieving behavioral choices over long-term, more helpful but delayed alternative behaviors [7].

In addition, researchers distinguish between self-report and neuropsychological measures of impulsivity and between the relationships of these measures to response inhibition and decision-making [4]. Also, Duckworth and Kern [8] state that in order to cover the various aspects of impulsivity, the symptom should be assessed by both self-report and task-related measures. Differences in overall neuropsychological variables between BPD patients and healthy individuals were also shown in recent meta-analyses, e.g [7]. For example, patients with BPD frequently exhibited deficits in areas of cognitive functioning, such as decision-making, attention, processing speed, or verbal intelligence, but not in overall intelligence [7].

Scores on self-report measures that reflect impulsivity as a personality trait, e.g., the Barratt Impulsiveness Scale (BIS-15 [9]), are usually higher in patients with BPD than in healthy individuals [4]. In addition, self-reported impulsiveness may predict treatment outcome [10]. In contrast, differences in impulsivity between patients with BPD and healthy controls is not often reflected in neuropsychological tasks [4]. These tasks use a behavioral paradigm that measures impulsivity by response inhibition, interference, cognitive control, delay discounting, and delay of gratification and assesses the potential interaction of these factors with emotional processing and regulation [4, 11]. Self-reported impulsivity tends to show a low correlation with delay discounting [12]. Furthermore, whereas response inhibition tasks such as go/no-go tasks usually show no difference between patients with BPD and healthy individuals, decision-making tasks such as the Iowa Gambling Task [13] usually indicate more risky behavior in BPD patients [4]. In addition, a meta-analysis by Paret et al. [14] also found significant differences in delay discounting tasks, e.g., the Monetary Choice Questionnaire (MCQ [15]), , between patients with BPD and healthy individuals. Delay discounting is an aspect of impulsivity in which the value of reward or reinforcement decreases the more distant or delayed the possible benefit is in the future [16]. Thus, this relationship is a hyperbolic function that uses the rate of discounting (indicated by the parameter ‘*k’*; [16]) to represent the subjective value of delayed rewards.

A common psychotherapeutic treatment for BPD is Dialectical Behavioral Therapy (DBT), which has proven its efficacy in many studies [17, 18]. In particular, Mungo et al. [3] showed that DBT is effective in treating impulsivity in BPD. DBT is a modularized therapeutic intervention that consists of several modules, including skills training, interpersonal skills, dealing with feelings, and mindfulness [19]. A key feature of DBT is behavioral analysis, which attempts to address the short- and long-term consequences of behavior and the associated alternative courses of action [20]. Therefore, as Cáceda et al. [11] stated, DBT focusses on the main characteristics of impulsive decision-making and delayed actions. In addition, Unoka and Richman [7] argue that BPD patients need to not only be made aware of the long-term consequences of impulsive behaviors, but also be shown alternative strategies for dealing with impulsive decisions. Hence, BPD patients should shift their focus of attention from present, immediate actions that could be harmful in the long term to autonomy-related decisions that may improve general functioning [7]. Interestingly, a recent study showed that DBT skills training improves impulsivity and decision-making also in alcohol and substance use disorder [21].

Neural correlates of impulsivity and decision-making are located in the prefrontal cortex, which consists of the orbitofrontal cortex, anterior cingulate cortex, and dorsolateral prefrontal cortex (DLPFC), which in turn are interconnected to other brain regions [5, 22]. In particular, the left DLPFC is associated with cognitive control and could be related to reward and risk procession [23]. These brain structures have been shown also to be impaired in patients with BPD [24–26]. Nevertheless, Van Zutphen et al. [25] also stated that in the case of impulsivity, more research is needed.

Nonpharmaceutical treatment approaches in mental disorders include repetitive transcranial magnetic stimulation (rTMS), a non-invasive brain stimulation (NIBS) method. rTMS is administered according to various stimulation protocols, including the recently introduced protocol of intermittent theta burst stimulation (iTBS), which increases neuronal excitability in the stimulated area [27]. The feasibility and efficacy of rTMS and iTBS in influencing decision-making have been shown in healthy participants [23, 28] and in patients with schizophrenia [29]. A meta-analysis by Yang et al. [30] showed that in healthy individuals, application of rTMS specifically on the left DLPFC modulated temporal decision-making. Regarding BPD, a review by Yahya et al. [31] suggested that rTMS could also lead to clinical improvements in impulsivity. Chiappini et al. [32] found that NIBS of the left DLPFC with approaches such as rTMS showed promising results in treating and augmenting the treatments in BPD. In their expert review [33], Lisoni et al. summarized the effects of NIBS, including rTMS, in the treatment of symptoms generally associated with BPD and included studies that also investigated the effect of NIBS on decision-making in individuals with BPD [34–36]; the authors concluded that NIBS may be a promising treatment option for BPD. One of the studies in their review found that when used to bilaterally stimulate the DLPFC, an NIBS intervention called transcranial direct current stimulation (tDCS) slightly influenced decision-making [34]. Furthermore, Calderón-Moctezuma et al. [35] showed that rTMS on the dorsomedial prefrontal cortex (DMPFC) led to improvements in decision-making tasks in BPD patients.

Concerning different facets of decision-making, Cailhol et al. found no effects of rTMS on risk-taking or impulsiveness but did find a significant improvement in planning abilities [36]. The preliminary results of Reyes López et al. [37] showed that after application of rTMS on either the left or right DLPFC, a range of BPD psychopathologic domains, including impulsiveness, decreased significantly in individuals with BPD. Additionally, a case series on three patients with BPD reported subjective improvement in impulse control after bilateral rTMS on the DMPFC [38]. Accordingly, rTMS and in particular iTBS may lead to improvements in impulsivity and decision-making in patients with BPD.

### Aims of the study

Because both DBT and rTMS may improve impulsivity and decision-making, we hypothesized that augmenting DBT with iTBS would lead to a greater reduction of impulsivity and greater improvement in decision-making than DBT alone. Furthermore, we assumed that over time, DBT per se would also improve decision-making. Thus, we expected that impulsivity measured by self-report would decrease over time and that decision-making as assessed by delay discounting would improve, i.e., that after treatment, patients would more frequently choose the larger, delayed reward rather than the smaller, immediate reward.

The data presented here represent a secondary analysis of a randomized controlled trial comparing iTBS add-on treatment with DBT in patients with BPD and comorbid depression who were receiving routine psychiatric inpatient treatment in Germany [39, 40]. Although the study did not find a significant effect of add-on iTBS treatment in various symptomatic measures (borderline or depressive symptoms) or social functioning, it showed a distinct trend in favor of iTBS (Cohen’s *d* = 0.23 for post-treatment group differences in borderline symptoms) and a significant main effect of DBT with and without iTBS (*d* = 0.89 to 1.12) [40].

## Method

### Procedure

Patients with BPD were recruited within the first 4 weeks of an 8- to 12-week routine inpatient DBT program on a specialized ward at the Department of Psychiatry, LVR Clinical Center Düsseldorf, Heinrich Heine University, Düsseldorf, Germany. Patients were given information about the study and all patients gave written informed consent by signing the declaration, patient information, and data protection documents. Subsequently, they were randomly assigned (1:1) to one of two groups: the DBT + active iTBS group, which received 20 sessions of iTBS, or the DBT + sham group, which received 20 sessions of sham stimulation. In both groups, stimulation was administered once daily, Monday to Friday, during weeks 5 to 8 of DBT treatment. Randomization was performed with MATLAB. The study was single-blinded, i.e., patients were unaware of their assigned condition, but study staff were not. Data analysis was performed blind to group allocation.

The trial study was registered in the German Clinical Trials Register (DRKS) on January 13, 2020, under the registration number DRKS00020413. It was approved by the ethics committee of the medical faculty at Heinrich-Heine-University, Düsseldorf, Germany, on December 13, 2019 (reference number: 2019 − 637), and protocol amendments were approved on July 27, 2020, and February 9, 2021. The study was conducted in accordance with all relevant laws, institutional guidelines, and the Code of Ethics of the World Medical Association (Declaration of Helsinki).

The routine inpatient DBT program was based on Bohus et al. [41] and adapted for use in the inpatient setting. The program comprises modules on skills training, emotion regulation, interpersonal skills, and mindfulness, and modules are performed weekly in individual and group therapy sessions. To ensure compliance with the DBT manual, all staff, including medical and nursing staff, psychotherapists, occupational therapists, and others, received training in all modules from the Dachverband DBT e.V. For further details, see Kujovic et al. [39].

### Inclusion and exclusion criteria

Patients were included if they met criteria for both BPD and comorbid major depression and had no other psychiatric comorbidities. Diagnoses were confirmed with the Diagnostic Interview for Mental Disorders (Mini-DIPS OA; [42]) and the Structured Clinical Interview for DSM-5 Personality Disorders (SCID-V-PD; [43]). Additional inclusion criteria were age 18 to 45 years, adequate German language skills, and ability to provide written informed consent. Participants on medication were required to maintain stable intake of therapeutic doses for at least two weeks prior to the start of the stimulation phase and throughout its four-week duration. Female participants required a negative pregnancy test and had to agree to use contraception throughout the study.

Participants were excluded if they had a history of epilepsy (seizures), metallic objects in the skull, extensive tattoos on the head, significant brain malformations or tumors, cerebrovascular events, traumatic brain injuries, neurodegenerative diseases, previous brain surgery, deep brain stimulation, other intracranial implants, a cardiac pacemaker, other severe physical illnesses, psychiatric conditions other than major depression and BPD, acute suicidal ideation (score > 4 on question 10 of the Montgomery-Åsberg Depression Rating Scale [44]), , tinnitus, pregnancy, claustrophobia, or current or past treatment with electroconvulsive therapy or vagus nerve stimulation. Patients were also excluded if they were taking anti-epileptic medications, including benzodiazepines, at doses equal to or exceeding 1 mg/day of lorazepam; were under legal guardianship with limited consent capacity; or had previously undergone DBT. According to the study protocol, patients who missed more than four iTBS or sham sessions were also excluded from the statistical analyses, as detailed in Kujovic et al. [39].

### iTBS and sham stimulation

The iTBS stimulation was administered with a PowerMAG Research 100 magnetic stimulator [45] equipped with a PMD70-pCool figure-of-eight coil; the stimulator was located on the psychiatric treatment ward. Before the initial treatment and two weeks thereafter, electromyography was used to automatically establish the resting motor threshold. The threshold was determined by integrating the motor evoked potential and using an algorithmic approach [46–48]. The left DLPFC was located by the Beam F3 method [49]. The stimulation intensity was set at 80% of the resting motor threshold. Each iTBS treatment session delivered a total of 600 stimuli, lasted three minutes and 12 s, and was characterized by intermittent stimulation involving two seconds of stimulation followed by an eight-second pause.

In the sham condition, a sham coil (PMD70-pCool-Sham [50]), was used to maintain patient blinding. This sham coil had comparable weight and emitted similar sounds to the iTBS coil, but it produced a lower magnetic field strength that only stimulated the immediate scalp region without affecting the brain. As a result, participants in the sham group experienced auditory and tactile sensations akin to those experienced by the active treatment group.

### Study endpoints

The main study outcomes were various symptom domains related to BPD, depression, and social functioning; these outcomes were reported in our previous publication [40]. The current study focused on impulsivity and decision-making, assessed by delay discounting. This secondary analysis was part of a larger study; whenever possible, to increase treatment adherence and reduce the dropout rate the larger study used shorter instruments if the reliability and validity of these instruments were equal to those of the respective longer instruments.

In line with Duckworth and Kern [8], the study used both a self-report and a task-related measure, i.e., the Barratt Impulsiveness Scale (BIS-15; [9]) and Monetary Choice Questionnaire (MCQ; [15, 16]), respectively. The BIS-15 is a self-report measure, which assesses impulsivity, has sufficient reliability and validity and has been validated in German [6]. In addition, in accordance with Meule et al. [51] the study analyzed the three subscales *non-planning impulsivity* (i.e., lack of future orientation or forethought), *motor impulsivity* (i.e., acting without thinking), and *attentional impulsivity* (i.e., inability to focus attention or concentrate).

The MCQ comprises 27 items that assess decision-making through delay discounting. It was chosen because it is straightforward to administer, is computer-based, and directly assesses delay discounting (a key aspect of impulsivity) through self-assessment of cognition and decisions involving immediate versus delayed rewards [52]. Consequently, participants have to choose between a smaller, immediate reward and a larger, delayed reward [16]. For example, they are asked to choose between €34 as an immediate reward or €35 in 186 days as a larger, delayed reward (the original version uses US dollars). Clinical studies have validated the MCQ in various disorders, e.g., addiction disorders [15]. We used the tool developed by Kaplan et al. [16] to calculate the *k* parameter, which indicates the degree of discounting and is derived from the pattern of choices that participants make throughout the experiment. Larger *k* parameters reflect higher impulsivity. To increase participants’ commitment and the attractiveness of the task, participants were able to actually win a proportionate reward from one of their 27 items. Thus, at the end of the experiment, one of the 27 conditions was randomly selected, and the patient was paid 10% of the chosen immediate or delayed reward (range: €2.20 to €17). Depending on whether the patient had chosen the immediate or delayed reward, the reward was paid out either immediately or sent to the patient after the chosen period (e.g., 186 days).

The BIS-15 and MCQ were assessed at the beginning and end of the four-week iTBS/sham treatment.

### Statistical analysis

We used a 2 × 2-factorial between-subjects design to assess differences in impulsivity (BIS-15) and decision-making (MCQ). Stimulation (ACTIVE vs. SHAM) was used as the between-subjects factor, and time as the within-subjects factor (T0 vs. T4). To address potential bias due to dropout, we performed a linear mixed model repeated measure (LMMRM) analysis with a heterogenous first-order autoregressive structure for the (variance-)covariance matrix. We performed post hoc tests to compare the two groups at each time point (to test in particular for group differences after treatment) and to examine time effects within each group. We calculated the LMMRM for the MCQ logarithmic-transformed mean score (which is recommended as the primary parameter for statistical analyses; [16]) and the MCQ mean score (as a sensitivity analysis and to facilitate interpretation). Each outcome variable was tested for normal distribution by performing a Kolmogorov-Smirnov test in each group separately. The original (raw) scores of the MCQ were not normally distributed in both groups (*p* < .001 respectively), whereas the logarithmic transformation as well as BIS data were normally distributed (each *p* > .20). Nevertheless, we conducted additional nonparametric analyses based on aligned rank transformed (ART) data as described by Wobbrock et al. [53]. As additional sensitivity analyses, we included the respective MCQ baseline score as a covariate. The significance level was set at an alpha value of 0.05. In addition, we calculated effect sizes, Cohen’s d, for group differences and time effects (each of which was based on differences in LMMRM-estimated means related to pooled observed standard deviations). All analyses were performed with IBM SPSS V29 [54].

## Results

A total of 53 patients were recruited into the study; 25 (47.2%) were randomized to the active iTBS stimulation and 28 (52.8%) to the sham stimulation. The respective CONSORT chart is included in the main publication on the primary outcomes [40]. In the active group, 8 patients (32.0%) were excluded because they received fewer than 16 stimulations, and in the sham group, 5 patients (17.9%) were excluded for the same reason and 4 additional patients (14.3%) were excluded because they did not complete any MCQs. The proportion of specific exclusions was not significantly different between the active and sham groups (*p* = .52). Accordingly, a total of *N* = 36 patients were included in the analysis, *n* = 17 in the active group (47.2%) and *n* = 19 in the sham group (52.8%). We tested whether excluded and included patients differed with respect to relevant variables (age, gender, education, illness duration, and baseline symptom scores), but we did not find any significant differences (see Supplementary Table S1).

Table 1 shows the baseline sample characteristics of the active and sham groups; 88.9% of patients were female (*n* = 32), and the mean age was 25.4 years (SD = 6.3). No group differences were found in symptom scores, but some were found in drug treatment (antidepressants and second-generation antipsychotics). The effect of the drug treatment differences on outcome was tested separately and was found to be non-significant (see Kujovic et al. [40]). The baseline BIS-15 and MCQ scores were not significantly different between the active and sham groups.

**Table 1:** Sample characteristics of patients included by treatment group

	SHAM (*n* = 19)	ACTIVE (*n* = 17)	Total (*N* = 36)	*p* ^1^
Age, mean (SD), y	25.9 (6.8)	24.8 (5.9)	25.4 (6.3)	0.58
Gender: female, n (%)	17 (89.5)	15 (88.2)	32 (88.9)	0.60
Handedness: right, n (%)	15 (78.9)	14 (82.4)	29 (80.6)	0.96
Smoking ‘yes’, n (%)	11 (57.9)	9 (52.9)	20 (55.6)	0.80
Years of education (school, university, or occupational training), mean (SD)	14.3 (2.2)	13.6 (2.4)	14 (2.3)	0.38
Employed, n (%)	11 (57.9)	7 (41.2)	18 (50.0)	0.51
Years after first BPD diagnosis, mean (SD)	1.5 (3.1)	2.0 (4.9)	1.7 (4.0)	0.72
BSL sum score, mean (SD)	44.6 (16.6)	39.9 (15.8)	42.4 (16.2)	0.39
BDI, mean (SD)	32.8 (10.2)	32.5 (12.4)	32.6 (11.2)	0.93
MADRS total score, mean (SD)	20.7 (5.1)	21.3 (5.6)	21 (5.3)	0.76
MADRS item 10 (suicidal thoughts), mean (SD)	1.3 (0.8)	1.3 (0.8)	1.3 (0.8)	0.94
SCS, mean (SD)	2.0 (0.7)	2.1 (0.5)	2.0 (0.6)	0.85
BIS-15, mean (SD)	39.1 (5.8)	40.0 (8.0)	39.5 (6.9)	0.69
- Non-planning impulsivity	12.9 (3.0)	13.0 (2.8)	13.0 (2.9)	0.93
- Motor impulsivity	13.5 (2.4)	13.7 (3.5)	13.6 (2.9)	0.81
- Attentional impulsivity	12.1 (2.8)	12.5 (2.6)	12.3 (2.7)	0.70
GAF, mean (SD)	54.5 (14.1)	52.3 (13.3)	53.5 (13.6)	0.63
MCQ, mean (SD)	0.03469 (0.0767)	0.01072 (0.01588)	0.02338 (0.05734)	0.20
MCQ_log_^2^, mean (SD)	-2.34222 (0.97617)	-2.63338 (0.8883)	-2.47971 (0.93409)	0.36
MCQ Consistency, mean (SD)	0.90253 (0.09737)	0.9281 (0.06207)	0.91461 (0.08249)	0.36
MCQ proportion ‘Late Delayed Reward’, mean (SD)	0.51267 (0.22504)	0.62309 (0.23286)	0.56481 (0.23229)	0.16
Psychotropic drugs, n (%)				
None	7 (36.8)	4 (23.5)	11 (30.6)	0.48
Any antidepressant	12 (63.2)	10 (58.8)	22 (61.1)	0.79
- **SSRI**	7 (36.8)	1 (5.9)	8 (22.2)	**0.044**
- SNRI	2 (10.5)	6 (35.3)	8 (22.2)	0.114
Any antipsychotic (AP)	2 (10.5)	5 (29.4)	7 (19.4)	0.22
- **Second generation AP**	0 (0)	4 (23.5)	4 (11.1)	**0.040**
Sum ACTIVE / SHAM sessions, mean (SD)	18.2 (1.5)	18.5 (1.3)	18.3 (1.4)	0.50
Resting motor threshold, mean (SD)	51.1 (12.9)	49.8 (11.0)	50.5 (11.9)	0.75
iTBS stimulus intensity, mean (SD)	40.8 (10.4)	39.6 (8.8)	40.2 (9.6)	0.72
Self-assessed treatment, n (%)				0.35
- unknown / unsure	4 (21.1)	2 (11.8)	6 (16.7)	
- SHAM	8 (42.1)	4 (23.5)	12 (33.3)	
- ACTIVE	7 (36.8)	11 (64.7)	18 (50.0)	

Active, intermittent theta burst stimulation; BDI, Beck’s Depression Inventory (second edition, BDI-II); BPD, Borderline Personality Disorder; BIS, Barratt Impulsiveness Scale (short form, BIS-15); BSL-23, 23-item Borderline Symptom List; GAF, Global Assessment of Functioning scale; MCQ, Monetary Choice Questionnaire; MADRS, Montgomery-Åsberg Depression Rating Scale; SCS, Self-Compassion Scale (German short version, SCS-D); Sham, sham stimulation; SSNRI, selective serotonin noradrenalin reuptake inhibitor; SSRI, selective serotonin reuptake inhibitor

^**1**^ Significance level for group differences; t test was used for continuous measures, Chi2 was used for frequencies / proportions, and exact testing was used in case of low cell frequencies

^2^ log = logarithmic transformation

The observed means and SDs of the MCQ and BIS-15 scores over time are shown for both groups in Supplementary Table S2. The BIS-15 results were analyzed by the LMMRM and are shown in Table 2. As can be seen, we found no differences in impulsivity between the active and sham groups over time (group*time interaction, *p* = .16). Nevertheless, impulsivity decreased over time in both groups (time main effect, *p* < .001). In post hoc tests, the mean time difference was significant in the active group (mean_diff_ = -4.7, *p* = .001, *d* = 0.68) but not in the sham group (mean_diff_ = -2.1, *p* = .077, *d* = 0.31). The analyses of the BIS subscales showed that this differential effect was particularly attributable to *motor impulsivity* (i.e., acting without thinking), where the group*time interaction reached borderline significance (*p* = .066, see also Table S4 in Supplement) and post hoc tests indicated a highly significant improvement in the active group (*p* = .005) but no significant change in the sham group (*p* = .58). For *non-planning impulsivity* (i.e., a lack of future orientation or forethought), the overall time effect was significant (*p* = .043), with marginal advantages for active (*p* = .08 in post hoc test) versus sham stimulation (*p* = .27; see also Table S3). In *attentional impulsivity* (i.e., the inability to focus attention or concentrate), both groups improved similarly (post hoc analysis of group-specific changes: active, *p* = .002; sham, *p* = .001; Supplementary Table S5).

**Table 2:** Results of mixed model repeated measure analysis of BIS-15

	Baseline (V0)		Post treatment (V4)			
	Estimated means	95% CI	Estimated means	95% CI	*p* ^**1**^	Effect size^**2**^
Sham	39.1	36.2 / 42.0	37.0	34.0 / 40.0	0.077	
Active	40.0	36.6 / 43.4	35.3	31.8 / 38.8	0.001	0.24

Results of mixed model repeated measures analysis: group, *p* = .84; time, *p* < .001; group*time, *p* = .16

BIS-15 = Barratt Impulsiveness Scale (short form)

^**1**^ Post hoc significance level for time effect within groups

^**2**^ Effect size (Cohen’s d) for group differences at V4

The results of the LMMRM analysis of the MCQ scores are shown in Table 3. The analysis showed no significant difference between the active and sham groups regarding the change in MCQ score over time (group*time interaction, *p* = .13). Also, the within-group difference over time was not significant for either group (time main effect, *p* = .32; sham: mean_diff_ = -0.007, *p* = .68, *d* = -0.15; active: mean_diff_ = 0.031, *p* = .095, *d* = 0.67). The results for the other MCQ scores are reported in the Supplement (Tables S6-S8) and were also not significant for the logarithmic-transformed MCQ score or for the models that included the baseline MCQ score as a covariate.

**Table 3:** Results of mixed model repeated measure analysis of MCQ

	Baseline (V0)		Post treatment (V4)			
	Estimated means	95% CI	Estimated means	95% CI	*p* ^**1**^	Effect size^**2**^
Sham	0.035	0.008 / 0.061	0.028	-0.005 / 0.061	0.68	
Active	0.011	-0.017 / 0.039	0.042	0.005 / 0.078	0.095	0.23

Results of mixed model repeated measures analysis: group, *p* = .77; time, *p* 0.32; group*time, *p* = .13

MCQ = Monetary Choice Questionnaire

^**1**^ Post hoc significance level for time effect within groups

^**2**^ Effect size (Cohen’s d) for group differences at V4

The results of additional nonparametric analyses based on ART data as described by Wobbrock et al. [52] corresponded to the parametric analyses described above and are shown in the Supplement (Tables S9 to S11).

## Discussion

In the present study, we investigated whether DBT with augmented iTBS has an enhancing effect on impulsivity and decision-making in patients with BPD. Contrary to our hypothesis, we found no significant difference between patients in the active and sham groups in the change in impulsivity, as measured by self-reported impulsivity (BIS-15) and neuropsychologically assessed decision-making regarding delay discounting (MCQ). Nevertheless, self-reported impulsivity decreased significantly over time in the active group, but not in the sham group.

To our knowledge, our study is the first to examine delay discounting in the context of DBT in BPD. Furthermore, iTBS is also a new area of research that has yet to demonstrate its effect on different neuropsychological variables. We found no additional effect of iTBS on delay discounting, perhaps because the aspects of decision-making controlled by the left DLPFC are different from the aspects measured by delay discounting. Also, the left DLPFC is not the only brain area associated with decision-making and impulsivity. Thus, a review by Brevet-Aeby et al. [55] found that stimulation of the left hemisphere and bilateral stimulation were studied less often. Their review also showed that stimulation of the right DLPFC, which modulates high-level cognitive processes and is associated with left/right hemispheric balance, is related to impulsivity [55]. Furthermore, differences between unilateral and bilateral stimulation and in the timing of stimulation play an important role when using NIBS in the treatment of impulsivity [55]. A case study by Svěrák et al. [56] applied rTMS on the right DLPFC in patients with BPD and showed that the stimulation enhanced emotion regulation and reduced impulsive behavior. Also, although Lisoni et al. [57] concluded that clinical evidence is still limited regarding rTMS and BPD, they emphasized the importance of the right DLPFC when studying impulsivity. A pilot study on tDCS by Lisoni et al. [34] that used bilateral stimulation and focused on the right DLPFC showed improvements in impulsivity and aggression in BPD patients. Therefore, future studies may also use unilateral and bilateral stimulation to examine the role of the DLPFC.

Because we did not find an additional effect of iTBS on delay discounting, we assume that this is a more complex phenomenon that needs to be further investigated. Delay discounting also did not decrease over time in either group. A possible explanation could be the MCQ condition we used for delay discounting: Patients were given the opportunity to receive a proportion of the money they chose in the decision tasks, so at baseline, they may have been biased to choose the larger, delayed amount because they expected to receive the money while they were still undergoing inpatient treatment, whereas at the second assessment at the end of treatment, they may have doubted whether they would receive the delayed reward after discharge, motivating them to more often select the immediate reward. In BPD, impulsivity is strongly associated with (high) emotional distress [1]. Consequently, within the assessment situation patients (mostly) did not feel discomfort or were in a (highly aversive) emotional state that did not involve impulsivity.

Although studies have shown a deficit in decision-making in BPD patients [14], to date few studies have investigated the effect of cognitive behavioral therapy on delay discounting. Furthermore, these studies often investigated substance abuse [58], which is associated with decision-making and impulsivity, but with only some aspects of BPD. Therefore, one could assume that aspects covered in DBT do not overlap with aspects measured by monetary delay discounting. In addition, the literature discusses whether delay discounting is a trait or is influenced by situational and contextual variables [58]. The former would make it much more difficult to address the issue, but the latter would suggest that it is possible to improve decision-making through training [58]. In a study by Scholten et al. [58], acceptance-/mindfulness-based training showed the most promising results. Acceptance and mindfulness are also covered in DBT, but they are only one aspect of it because the therapy focuses primarily on self-harming behaviors. Furthermore, decision-making is believed to involve many different components, making it a difficult topic to work on with patients [58].

The BIS-15 showed no differences in self-reported impulsivity between the active and sham groups. Although impulsivity decreased over time in both groups, the change was significant only in the active group. In contrast to delayed discounting measured by the MCQ, self-assessed impulsivity measured with the BIS-15 covers a broader timespan and also includes more stressful or emotional situations. Thus, the reductions in impulsivity as measured by the BIS-15 may reflect an effect of both DBT and additional iTBS on impulsivity, suggesting that the combination of DBT and iTBS should be further investigated.

The BIS-15 is primarily a self-report measure that assesses the domains of *non-planning impulsivity*, *motor impulsivity*, and *attentional impulsivity*, and it is thought to measure a personality trait. Regarding the BIS-15 subscales, the analyses showed that the significant decrease in impulsivity over time, which was found in the active but not in the sham group, was attributable to differences in changes in *non-planning impulsivity* and *motor impulsivity*. Changes in *attentional impulsivity* were significant in both groups.

Accordingly, although some items reflect a type of impulsivity that is also found in other mental disorders (e.g., the item “I am restless in the theatre or in lectures”, which would be in line with attention deficit hyperactivity disorder) and may be less amenable to change, other items assess whether participants see themselves as being able to plan for the future or for job security and as tending to spontaneously buy things, which is associated with emotional dysregulation. Therefore, most psychotherapeutic interventions address some of these items, and the items may improve over time as strategies for coping with future challenges are developed. Also unclear from the literature is whether DBT or iTBS directly influences impulsivity because this question depends on how impulsivity is defined and measured. For example, a meta-analysis by Stoffers-Winterling et al. [17] found that standard DBT had no effect on impulsivity, whereas DBT with a focus on group skills training did. Also, complex symptoms such as impulsivity appear to be more resistant to change [59]. As stated above, mindfulness training may be helpful in delay discounting, a study by Soler et al. [60] found no positive effect on self-reported impulsivity in BPD patients undergoing DBT. More research is needed on rTMS and iTBS regarding delay discounting and impulsivity. Nevertheless, as a strength of the current study, we chose to use a self-report and a task measure to assess impulsivity and decision-making, as these have some, albeit heterogeneous, convergent validity [8]. Hence, various facets of impulsivity and decision-making may necessitate distinct interventional approaches.

### Limitations

The study has some limitations. First, the sample was small because the study was designed as a pilot study, therefore, it may have been too small to detect a significant effect. Second, the study was single blinded, so future research should use a double-blind study design; however, we tried to address this issue by performing blinded data analysis and by asking patients whether they could guess which group they were allocated to. Third, strict inclusion and exclusion criteria may lead to selection bias and limit external validity. Fourth, the study had no follow-up, so we cannot draw any conclusions about long-term effects. Fifth, we focused on emotion dysregulation as a major symptom of BPD and therefore stimulated the left DLPFC, but future studies could focus also on other brain areas. Sixth, the patients in this study all met the inclusion criteria of a comorbid depression. Because comorbid depression may delay the remission of BPD symptoms [61], future studies should investigate whether results are different in BPD patients with and without depression. Last, we applied stimulation after patients had completed 4 weeks of DBT, so future studies should further investigate the timing of iTBS as an add-on to DBT.

## Conclusion

Although we did not find differences between the groups, augmentation of DBT or other psychotherapeutic interventions with brain stimulation approaches remains a promising approach. Furthermore, impulsivity and decision-making in BPD should be further investigated. We suggest large, longitudinal double-blind studies for this purpose.

## Electronic supplementary material

Below is the link to the electronic supplementary material.

Supplementary Material 1

## Data Availability

Data will be provided to any researcher by the corresponding author upon reasonable request.
