# Supplementary material for: Effects on impulsivity and delay discounting of intermittent theta burst stimulation add-on to dialectical behavioral therapy in borderline personality disorder: a randomized, sham-controlled pilot trial
Source: Borderline Personal Disord Emot Dysregul. 2025 Jan 14;12:2. doi: 10.1186/s40479-025-00278-3 (PMC11734458; doi:10.1186/s40479-025-00278-3)
Supplement: Supplementary file 1 — Supplementary Material 1 [file 40479_2025_278_MOESM1_ESM.docx]

**Supplementary**

Table S1: Characteristics of patients dropping-out or being excluded (<16 stimulation sessions or missing MCQ score) vs. included patients

|  | **Excluded (n = 17)** | **Included (n = 36)** | **Total (N = 53)** | **p^1^** |
| --- | --- | --- | --- | --- |
| Age, mean (SD), y | 24.9 (5.8) | 25.4 (6.3) | 25.2 (6.1) | 0.78 |
| Gender: female, n (%) | 14 (82.4) | 32 (88.9) | 46 (86.8) | 0.38 |
| Height, mean (SD), cm | 167.8 (9.2) | 169.6 (6.7) | 169 (7.6) | 0.43 |
| Weight, mean (SD), kg | 70.7 (17.2) | 78.9 (21.1) | 76.3 (20.1) | 0.17 |
| BMI, mean (SD) | 25.0 (5.0) | 26.9 (6.2) | 26.3 (5.8) | 0.28 |
| Education (school, university or job), mean (SD), y | 13.1 (2.5) | 14.0 (2.3) | 13.7 (2.4) | 0.21 |
| MADRS total score, mean (SD) | 23.8 (8.2) | 21.0 (5.3) | 21.9 (6.4) | 0.22 |
| MADRS item 10 (suicidal thoughts), mean (SD) | 1.9 (1.2) | 1.3 (0.8) | 1.5 (1.0) | 0.08 |
| BSL, mean (SD) | 39.1 (19.7) | 42.4 (16.2) | 41.4 (17.2) | 0.54 |
| BDI, mean (SD) | 31.9 (12.9) | 32.6 (11.2) | 32.4 (11.6) | 0.84 |
| SCS, mean (SD) | 2.1 (0.7) | 2.0 (0.6) | 2.1 (0.6) | 0.71 |
| BIS-15, mean (SD) | 38.9 (6) | 39.5 (6.9) | 39.3 (6.6) | 0.75 |
| GAF, mean (SD) | 50.8 (6.3) | 53.5 (13.6) | 52.7 (11.8) | 0.46 |
| Years after first BPD diagnosis, mean (SD) | 1.6 (2.1) | 1.7 (4) | 1.7 (3.5) | 0.89 |
| Randomized treatment, n (%) - SHAM - ACTIVE | 9 (52.9) 8 (47.1) | 19 (52.8) 17 (47.2) | 28 (52.8) 25 (47.2) | 0.99 |

Active, intermittent theta burst stimulation; BDI, Beck’s Depression Inventory (second edition, BDI-II); BPD, Borderline Personality Disorder; BIS, Baratt Impulsiveness Scale (short form, BIS-15); BSL-23, 23-item Borderline Symptom List; GAF, Global Assessment of Functioning scale; MADRS, Montgomery-Åsberg Depression Rating Scale; MCQ, Monetary Choice Questionnaire; SCS, Self-Compassion Scale (German short version, SCS-D); Sham, sham stimulation.

**^1^** Significance level for group differences; t test was used for continuous measures, Mann-Whitney test was used if normal distribution was not given (applies only to “Years since first BPD diagnosis”), and Chi2 was used for frequencies / proportions

Table S2: Treatment course of observed values in various outcome measures by treatment groups

|  | **sham** | **sham** | **sham** | **active** | **active** | **active** |
| --- | --- | --- | --- | --- | --- | --- |
| **Outcome by time** | **N** | **mean** | **SD** | **N** | **mean** | **SD** |
| **MCQ mean** |  |  |  |  |  |  |
| 0 | 19 | 0.03469 | 0.0767 | 17 | 0.01072 | 0.01588 |
| 4 | 19 | 0.02811 | 0.0566 | 15 | 0.04206 | 0.08579 |
| Change 0 to 4 | 19 | -0.00658 | 0.0465 | 15 | 0.02993 | 0.09183 |
| **MCQ_log_ ^1^** |  |  |  |  |  |  |
| 0 | 19 | -2.34222 | 0.97617 | 17 | -2.63338 | 0.8883 |
| 4 | 19 | -2.0256 | 0.64158 | 15 | -2.18752 | 0.85094 |
| Change 0 to 4 | 19 | 0.31662 | 0.84365 | 15 | 0.29019 | 1.38618 |
| **MCQ consistency** |  |  |  |  |  |  |
| 0 | 19 | 0.90253 | 0.09737 | 17 | 0.9281 | 0.06207 |
| 4 | 19 | 0.92203 | 0.10966 | 15 | 0.93827 | 0.0836 |
| **MCQ proportion of 'Late Delayed Reward'** |  |  |  |  |  |  |
| 0 | 19 | 0.51267 | 0.22504 | 17 | 0.62309 | 0.23286 |
| 4 | 19 | 0.46589 | 0.1721 | 15 | 0.50123 | 0.21816 |
| **BIS-15** |  |  |  |  |  |  |
| 0 | 19 | 39.1 | 5.8 | 17 | 40.0 | 8.0 |
| 4 | 19 | 37.0 | 6.1 | 17 | 35.3 | 8.3 |
| - Non-planning impulsivity |  |  |  |  |  |  |
| 0 | 19 | 12.9 | 3.0 | 17 | 13.0 | 2.8 |
| 4 | 19 | 12.3 | 2.7 | 17 | 11.9 | 2.8 |
| - Motor impulsivity |  |  |  |  |  |  |
| 0 | 19 | 13.5 | 2.4 | 17 | 13.7 | 3.5 |
| 4 | 19 | 13.2 | 2.9 | 17 | 11.8 | 3.3 |
| - Attentional impulsivity |  |  |  |  |  |  |
| 0 | 19 | 12.1 | 2.8 | 17 | 12.5 | 2.6 |
| 4 | 19 | 10.6 | 2.8 | 17 | 10.8 | 3.2 |

Active, intermittent theta burst stimulation; BIS-15 = Baratt Impulsiveness Scale; MCQ, Monetary Choice Questionnaire; Sham, sham stimulation; Non-planning impulsivity (lack of future orientation or forethought); Motor impulsivity (acting without thinking); Attentional impulsivity (inability to focus attention or concentrate)

^1^ log = logarithmic transformation

Table S3: Results of mixed model repeated measure analysis of BIS-15 sub-scale 'non-planning impulsivity' (lack of future orientation or forethought)

|  | Baseline (V0) | | Post treatment (V4) | |  |
| --- | --- | --- | --- | --- | --- |
|  | Estimated means | 95% CI | Estimated means | 95% CI | *p***^1^** |
| Sham | 12.9 | 11.7 - 14.1 | 12.3 | 11.1 - 13.5 | 0.27 |
| Active | 13.0 | 11.6 - 14.4 | 11.9 | 10.5 - 13.2 | 0.08 |

Results of mixed model repeated measures analysis: group: *p* = .84; time: *p* = .043; group*time: *p* = .54

**^1^** Post hoc significance level for time effect within groups

Table S4: Results of mixed model repeated measure analysis of BIS-15 sub-scale 'motor impulsivity' (acting without thinking)

|  | Baseline (V0) | | Post treatment (V4) | |  |
| --- | --- | --- | --- | --- | --- |
|  | Estimated means | 95% CI | Estimated means | 95% CI | *p***^1^** |
| Sham | 13.5 | 12.2 - 14.7 | 13.2 | 11.9 - 14.5 | 0.58 |
| Active | 13.7 | 12.3 - 15.1 | 11.8 | 10.3 - 13.3 | 0.005 |

Results of mixed model repeated measures analysis: group: *p* = .52; time: *p* = .013; group*time: *p* = .066

**^1^** Post hoc significance level for time effect within groups

Table S5: Results of mixed model repeated measure analysis of BIS-15 sub-scale 'attentional impulsivity' (inability to focus attention or concentrate)

|  | Baseline (V0) | | Post treatment (V4) | |  |
| --- | --- | --- | --- | --- | --- |
|  | Estimated means | 95% CI | Estimated means | 95% CI | *p***^1^** |
| Sham | 12.1 | 11.0 - 13.3 | 10.6 | 9.4 - 11.9 | 0.001 |
| Active | 12.5 | 11.1 - 13.8 | 10.8 | 9.4 - 12.3 | 0.002 |

Results of mixed model repeated measures analysis: group: *p* = .75; time: *p* < .001; group*time: *p* = .85

**^1^** Post hoc significance level for time effect within groups

Table S6: Results of mixed model repeated measure analysis of MCQ_log_^1^

|  | Baseline (V0) | | Post treatment (V4) | |  |  |
| --- | --- | --- | --- | --- | --- | --- |
|  | Estimated means | 95% CI | Estimated means | 95% CI | p^2^ | Effect size**^2^** |
| Sham | -2.34 | -2.78 / -1.91 | -2.03 | -2.37 /-1.68 | 0.23 |  |
| Active | -2.63 | -3.10 /-2.17 | -2.20 | -2.59 /-1.81 | 0.13 | 0.23 |

Results of mixed model repeated measures analysis: group: p = .28; time: p .059; group*time: p = .76

^1^ log = logarithmic transformation

**^2^** Post hoc significance level for time effect within groups

**^3^** Effect size (Cohen's d) for group differences at V4

Table S7: Results of mixed model analysis of MCQ including MCQ baseline score as covariate

|  | Baseline (V0) | | Post treatment (V4) | |  |  |
| --- | --- | --- | --- | --- | --- | --- |
|  | Estimated means | 95% CI | Estimated means | 95% CI | p^2^ | Effect size**^2^** |
| Sham | 0.025^3^ |  | 0.023 | -0.008 / 0.054 | 0.89 |  |
| Active | 0.025^3^ |  | 0.049 | 0.014 / 0.083 | 0.17 | 0.37 |

Results of mixed model repeated measures analysis: MCQ baseline score: p = .013; group. p = .27

**^1^** Post hoc significance level for time effect within groups

**^2^** Effect size (Cohen's d) for group differences at V4

^3^ Constant score estimated by mixed model repeated measures analysis

Table S8: Results of mixed model analysis of MCQ_log_^1^ including MCQ baseline score as covariate

|  | Baseline (V0) | | Post treatment (V4) | |  |  |
| --- | --- | --- | --- | --- | --- | --- |
|  | Estimated means | 95% CI | Estimated means | 95% CI | p^2^ | Effect size**^2^** |
| Sham | -2.40^4^ |  | -2.03 | -2.38 / -1.68 | 0,04 |  |
| Active | -2.40^4^ |  | -2.18 | -2.58 / 1.79 | 0,26 | 0.20 |

Results of mixed model repeated measures analysis: MCQ baseline score: p = .56; group. p = .57

^1^ log = logarithmic transformation

**^2^** Post hoc significance level for time effect within groups

**^3^** Effect size (Cohen's d) for group differences at V4

^4^ Constant score estimated by mixed model repeated measures analysis

Table S9: Results of mixed model analysis based on aligned rank transformed data (ART) **for BIS**

| **Effect** | **F** | **p^1^** |
| --- | --- | --- |
| Group | 0 | 0.98 |
| Time | 12.91 | 0.001 |
| Group * Time | 1.17 | 0.29 |

^1^ Significance level for main and interaction effects

Table S10: Results of mixed model analysis based on aligned rank transformed data (ART) **for MCQ**

| **Effect** | **F** | **p^1^** |
| --- | --- | --- |
| Group | 0.13 | 0.72 |
| Time | 0.44 | 0.51 |
| Group * Time | 0.15 | 0.70 |

^1^ Significance level for main and interaction effects

Table S11: Results of mixed model analysis based on aligned rank transformed data (ART) **for MCQ_log_**^1^

| **Effect** | **F** | **p^1^** |
| --- | --- | --- |
| Group | 1.37 | 0.25 |
| Time | 3.97 | 0.055 |
| Group * Time | 0.003 | 0.95 |

^1^ Significance level for main and interaction effects
